# Single-Cell Cloning and Transcriptomic Analysis Support a Myogenic Origin of Bovine Intramuscular Adipocytes

**DOI:** 10.3390/cells14221807

**Published:** 2025-11-18

**Authors:** Zhendong Tan, Binod Pokhrel, Honglin Jiang

**Affiliations:** School of Animal Sciences, Virginia Tech, Blacksburg, VA 24061, USA; zdtan93@vt.edu (Z.T.); drbinod@vt.edu (B.P.)

**Keywords:** adipogenic, intramuscular fat, myogenic, preadipocytes, subcutaneous fat

## Abstract

Intramuscular fat (IMF) refers to the adipose tissue located between muscle fibers and is a major determinant of meat quality in cattle. The cellular origin of bovine intramuscular adipocytes remains unclear. Therefore, the objective of this study was to investigate this origin. We derived single-preadipocyte clones from IMF and subcutaneous fat (SF) of cattle through single-cell cloning and subsequent validation of their potential to differentiate into adipocytes. Transcriptomic analysis of selected single-preadipocyte clones revealed that although both IMF- and SF-derived preadipocyte clones expressed classical preadipocyte markers such as *PDGFRA*, *DLK1*, and *ZNF423*, they differed significantly in global gene expression profile. Notably, many muscle-specific genes (e.g., *MYOG*, *MB*, and *MYH3*) were expressed at high levels in IMF-derived preadipocyte clones while not expressed in SF-derived clones. Functional enrichment analysis of differentially expressed genes between IMF- and SF-derived preadipocyte clones indicated that many muscle-related functions were enriched in the former. Furthermore, high-level expression of muscle-specific genes persisted in mature adipocytes differentiated from IMF-derived preadipocyte clones. We also found that bovine satellite cells, the widely considered progenitor cells of myocytes in postnatal animals, had the ability to form both myocytes and adipocytes under respective differentiation conditions. Based on these findings, we conclude that in cattle, at least some intramuscular adipocytes are derived from satellite cells.

## 1. Introduction

In cattle and other meat-producing animals, the function of adipose tissue varies with its location in the body. Among different fat depots, intramuscular fat (IMF) plays a particularly important role in determining the quality and economic value of beef, as it enhances beef flavor, juiciness, and tenderness [1]. In contrast, subcutaneous fat (SF) primarily functions as an energy reservoir and plays a role in systemic metabolic regulation; its contribution to meat quality is limited, and excessive SF deposition actually has a negative impact on carcass yield and processing efficiency [2,3]. Beyond these economic considerations, IMF and SF exhibit differences in proteomic profiles [4], morphological structures [5], lipid composition [6], metabolic activity [7], and cellular development [8].

The stromal vascular fraction (SVF) of adipose tissue is a heterogeneous cell population comprising different cell types that perform different functions [9]. In recent years, SVF cells have gained significant attention in regenerative medicine due to their containing multipotent stem cells that can support tissue repair and regeneration [9]. More importantly, SVF cells contain preadipocytes, cells that are committed from adipose progenitor cells (APCs) and are the immediate precursor cells for adipocytes [10]. Traditionally, SVF is obtained by enzymatically digesting adipose tissue, followed by centrifugation [11].

Compared to our understanding of how preadipocytes differentiate into adipocytes [12], little is known about the identity and origin of preadipocytes in various fat depots [13]. Intramuscular fat, being closely associated with muscle tissue, exists in a more complex microenvironment compared to SF [14]. It is believed that during early embryonic development, myocytes, adipocytes, and fibroblasts originate from a common progenitor pool, suggesting that the commitment of APCs to preadipocytes in IMF follows a more intricate pathway. Several cell types, including muscle satellite cells [15], fibroblasts associated with perimysial connective tissue [16], and fibro-adipogenic progenitor (FAP) cells [17], have been proposed as potential precursor cells for preadipocytes within the muscle.

Given these uncertainties, our study aimed to characterize the molecular signatures of preadipocyte populations in bovine IMF and SF tissues. Combining single-cell cloning, transcriptomic profiling, and differentiation assays, we found that IMF preadipocytes are different from SF preadipocytes in gene expression profiles and adipogenic potential. Our findings reveal that in cattle, intramuscular and subcutaneous preadipocytes have distinct origins. Specifically, intramuscular preadipocytes may derive from satellite cells, which are widely regarded as myogenic progenitor cells in postnatal animals.

## 2. Materials and Methods

### 2.1. Chemicals and Reagents

Unless otherwise indicated, all chemicals and reagents used in this study were purchased from Thermo Fisher Scientific Inc. (Waltham, MA, USA).

### 2.2. Animals and Collection of Tissue Samples

The experimental use of animals in this project received approval from the Institutional Animal Care and Use Committee at Virginia Tech (IACUC #20-169). The *longissimus dorsi* muscle samples (~100 g each) and subcutaneous fat samples (~10 g) were collected from six Angus-crossbred steers. These animals were slaughtered at approximately 18 months of age. For the final 6 months prior to slaughter, the steers were finished on a high-energy diet consisting of 11.3 kg of corn silage, 2.3 kg of cracked corn, and 3.2 kg of corn gluten per day to enhance growth performance and adipose tissue deposition. All animals were group-housed in open pens with ad libitum access to fresh water and shade. Tissue samples were transported to the laboratory on ice in pre-chilled phosphate-buffered saline (PBS). Skeletal muscle samples were used for IMF, SVF, and satellite cell isolation. Subcutaneous fat samples were used for SVF isolation. In the lab, pieces of IMF were carefully dissected from skeletal muscle, and any visible muscle attached to IMF was diligently removed with scissors.

### 2.3. Isolation of Stromal Vascular Fraction

Stromal vascular fractions from six IMF or SF samples were individually isolated as described previously [11]. Briefly, adipose tissue was washed with PBS and then cut into pieces of approximately 1 mm^3^. The tissue pieces were digested with type I collagenase at 2 mg/mL for 1 h at 37 °C in a shaking incubator. The dissociated cells were filtered through a 100 μm cell strainer and centrifuged at 500× *g* for 15 min. The cell pellet was resuspended and incubated with 5 mL 1× red blood cell lysis buffer for 5 min at room temperature to remove residual erythrocytes, followed by another centrifugation. The isolated SVF cells were either cryopreserved or cultured in growth medium containing Dulbecco’s modified eagle medium (DMEM), 10% fetal bovine serum (FBS, Phoenix Scientific, Candler, NC, USA), 2 mM L-glutamine, and 1% antibiotics–antimycotics (ABAM) at 37 °C with 5% CO_2_.

### 2.4. Isolation of Satellite Cells

Bovine satellite cells (bSCs) were isolated using a procedure as described before [15]. Briefly, the skeletal muscle samples were washed three times with PBS to remove blood, and any visible fat and connective tissue associated with the muscle were carefully trimmed. The cleaned muscle was minced with a meat grinder and then digested with 1 mg/mL pronase and 1 mg/mL collagenase type I for 1 h at 37 °C in a shaking incubator. The digestion was centrifuged at 400× *g* for 5 min to remove undigested tissues, and the supernatant was collected. The supernatant was then centrifuged at 1500× *g* for 10 min to pellet cells. This sequential centrifugation process (400× *g* followed by 1500× *g*) was repeated three additional times to enhance the yield of bSCs. Upon isolation, bSCs underwent a 5 min preplating procedure to improve their purity [18]. Bovine satellite cells were either cryopreserved or cultured in growth medium consisting of DMEM, 10% FBS, 2 mM L-glutamine, and 1% ABAM.

### 2.5. Single-Cell Cloning

Cloning of single SVF cells was performed by the limiting dilution method as described before [19]. The isolated SVF cells were diluted to a concentration of 5 cells/mL in growth medium, and 100 μL of the diluted suspension was plated into each well of 96-well plates using a multichannel pipette, aiming for 0 or 1 cell per well. Plates were cultured at 37 °C with 5% CO_2_ for approximately 10 days, with medium changed every 2 days. During this 10-day culture, wells were carefully scanned with an inverted microscope (Olympus Corporation, Tokyo, Japan) for the formation of single-cell colonies, which were then marked and transferred to 48-well plates for expansion. Single SVF-derived colonies were split at a ratio of 1:3 and continued to be expanded in growth medium in 24-well plates. When cells reached approximately 80% confluence, they were collected for RNA sequencing (RNA-seq) and other assays as described below.

### 2.6. Adipogenic Differentiation Assay

To induce adipogenic differentiation, SVF cells or bSCs were cultured in growth medium until reaching 100% confluence. Cells were maintained in growth medium for 2 additional days and then cultured in differentiation medium composed of DMEM/F12, 10 μg/mL bovine insulin (Sigma-Aldrich, St. Louis, MO, USA), 0.25 μM dexamethasone (Sigma-Aldrich), 0.5 mM 3-isobutyl-1-methylxanthine (IBMX, Sigma-Aldrich), 1 μM rosiglitazone (Cayman Chemical, Ann Arbor, MI, USA), and 1% ABAM for 2 days. Subsequently, cells were cultured in maintenance medium containing DMEM/F12, 10% FBS, 10 μg/mL bovine insulin, 1 μM rosiglitazone, and 1% ABAM for 4 additional days, during which maintenance medium was refreshed once. Cells were then subjected to Oil Red O staining or RNA extraction.

### 2.7. Myogenic Differentiation Assay

For myogenic differentiation, bSCs were cultured in growth medium to reach approximately 80% confluence and then cultured in myogenic differentiation medium consisting of DMEM, 2% horse serum (R&D Systems, Minneapolis, MN, USA), and 1% ABAM for 6 days, during which medium was refreshed every two days.

### 2.8. Oil Red O Staining

Cells were washed twice with PBS and then fixed with 10% neutral buffered formalin (Sigma-Aldrich) at room temperature for 1 h. After fixation, cells were rinsed with PBS and stained with Oil Red O solution (3.5 mg/mL in 60% isopropanol) for 15 min. Following staining, cells were gently washed with distilled water to remove excess dye and subsequently photographed with an OLYMPUS DP74 color digital camera and an OLYMPUS CKX41 inverted microscope (Olympus Corporation, Tokyo, Japan).

### 2.9. RNA Sequencing and Bioinformatic Analyses

Total RNA was extracted using the Direct-zol RNA MiniPrep Plus Kit (Zymo Research, Irvine, CA, USA), following the manufacturer’s instructions. RNA concentrations were quantified and purity assessed using a NanoDrop One spectrophotometer. All eight RNA samples used for RNA-seq had an RNA integrity number (RIN) greater than 7. RNA library construction and sequencing were performed by Novogene Corporation (Sacramento, CA, USA). Non-stranded RNA-seq libraries were prepared using the mRNA-Seq Library Preparation V2 Kit (ABclonal, Woburn, MA, USA). Libraries from four IMF and four SF preadipocyte clones were pooled and sequenced on an Illumina NovaSeq 6000 platform to generate paired-end reads.

RNA-seq data analyses, including quality control, trimming, alignment, and gene expression quantification, were carried out as previously described [20]. Briefly, low-quality reads and adapter sequences were removed using Trimmomatic v0.39 [21]. High-quality clean reads were aligned to the *Bos taurus* ARS-UCD2.0 reference genome using HISAT2 [22]. Genomic annotation files in GFF format were converted to GTF format using Gffread v0.12.18 [23]. Gene expression levels were quantified as transcripts per million (TPM) using FeatureCounts (v2.0.2) [24]. Genes with total read counts below 20 across all samples were filtered out to minimize noise and improve analytical accuracy. Differential gene expression analysis was performed with DESeq2 (v1.43.1) in R [25], using a stringent threshold of adjusted *p*-value < 0.05 and |log2 fold change| ≥ 1 to identify differentially expressed genes (DEGs). Functional enrichment analyses, including Gene Ontology (GO) and Kyoto Encyclopedia of Genes and Genomes (KEGG) pathway analyses, were conducted using the ClusterProfiler (v4.10.0) R package [26].

### 2.10. Reverse Transcription-Quantitative PCR (RT-qPCR)

First-strand cDNA was synthesized using random primers and the ImProm-II reverse transcription system (Promega, Madison, WI, USA). The qPCR was performed in duplicate using the Power SYBR Green PCR Fast Master Mix (Applied Biosystems, Foster City, CA, USA) on an Applied Biosystems 7500 Real-Time PCR System. The thermal cycling conditions were 95 °C for 20 s, followed by 40 cycles of 95 °C for 3 s and 60 °C for 30 s. Primer sequences (Table 1) were designed using the online NCBI Primer-BLAST tool. Gene expression levels were calculated using the 2^−ΔΔCt^ method [27], with *HMBS* serving as the internal control. The *HMBS* gene was chosen as the internal control because its expression was stable between the experimental groups in this study.

### 2.11. Statistical Analysis

All statistical analyses were conducted using JMP Pro v16.0.0 (SAS, Cary, NC, USA). Comparisons between two groups (IMF vs. SF) were performed using Student’s *t*-test. For comparisons among multiple groups, one-way ANOVA was applied, followed by Tukey’s post hoc test. All data are presented as mean ± SEM, with *n* = 4 for RNA-seq data and *n* = 6 for other data. Statistical significance was defined at *p* < 0.05.

## 3. Results

### 3.1. Cloning of Single Intramuscular and Subcutaneous Preadipocytes

**Figure 1:**
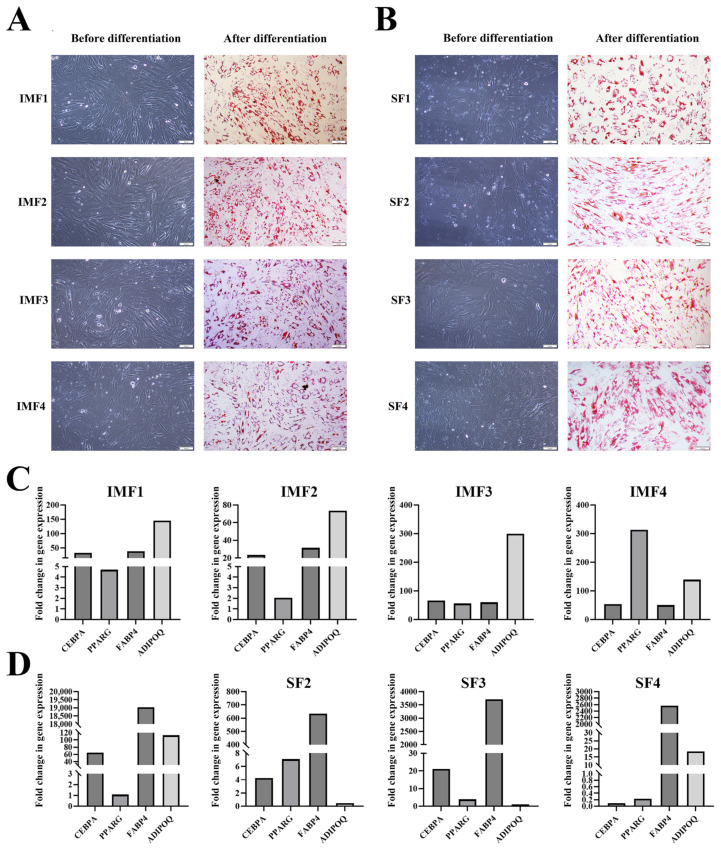
Differentiation of single-cell clones derived from stromal vascular fraction (SVF) of intramuscular fat (IMF) or subcutaneous fat (SF) into adipocytes upon adipogenic differentiation. (**A**,**B**) Oil Red O staining showing accumulation of lipid droplets in adipocytes differentiated from four single-cell clones derived from IMF (**panel A**) or SF (**panel B**) SVF. Each pair of images in (**panel A** or **B**) represent the same clone before and after 6-day adipogenic differentiation. Scale bar: 50 μm. (**C**,**D**) Increased expression of selected adipocyte marker genes in adipocytes differentiated from single-cell clones derived from IMF (**panel C**) or SF (**panel D**) SVF. Fold change is calculated by dividing the gene expression level after differentiation by that immediately before differentiation.

Single-cell cloning yielded a total of 48 clones from four IMF SVF samples and 70 clones from four SF SVF samples. Of these, 16 IMF SVF clones and 18 SF SVF clones exhibited adipogenic differentiation potential, as evidenced by successful formation of lipid droplets upon adipogenic induction. These clones were therefore considered preadipocyte clones. Four preadipocyte clones (IMF1-4) each from an IMF SVF sample and four preadipocyte clones (SF1-4) each from a SF SVF sample were selected for subsequent transcriptomic and functional analyses, based on their relatively high proliferative rate, relatively strong adipogenic potential, and fibroblast-like morphology. All 8 clones retained the capacity to differentiate into adipocytes, as evidenced by significant accumulation of lipid droplets (Figure 1A,B) and upregulation of adipocyte marker genes, including *PPARG*, *CEBPA*, *FABP4*, and *ADIPOQ*, after 6-day adipogenic differentiation compared to before adipogenic differentiation (Figure 1C,D).

### 3.2. Different Gene Expression Profiles Between Intramuscular and Subcutaneous Preadipocyte Clones

RNA-seq libraries were constructed from four IMF and four SF preadipocyte clones mentioned above. Sequencing these libraries generated a total of 384 million high-quality reads, with individual libraries yielding between 41.6 million and 65.3 million reads (Table S1). On average, 93.9% of these reads (ranging from 93.4% to 94.3%) successfully mapped to the bovine genome, with 91.0% (ranging from 90.6% to 91.7%) uniquely mapped (Table S1).

**Figure 2:**
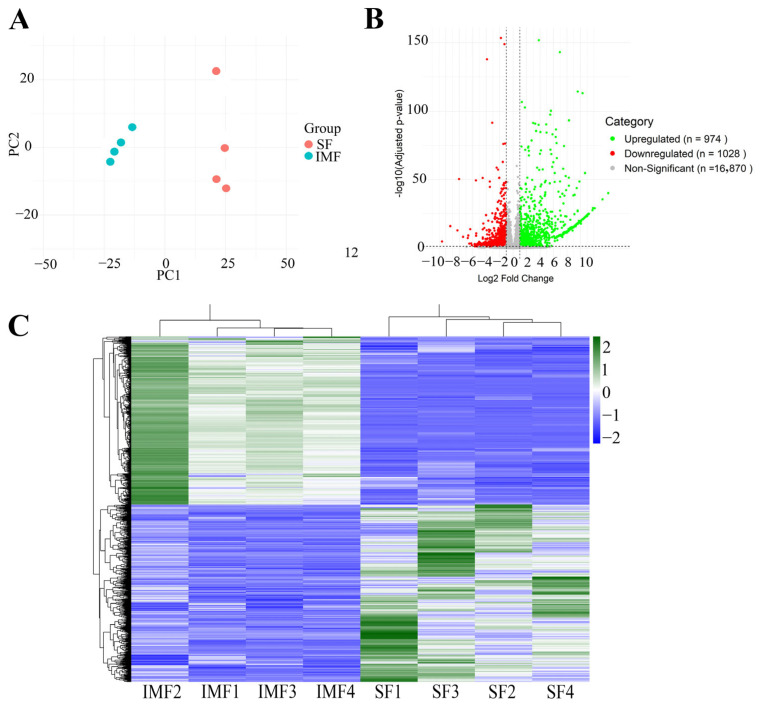
Transcriptomic analysis of single-preadipocyte clones derived from stromal vascular fraction (SVF) of intramuscular fat (IMF) and subcutaneous fat (SF) by RNA sequencing. (**A**) Principal component analysis (PCA) showing clear separation of gene expression profiles between four IMF single-preadipocyte clones and four SF single-preadipocyte clones. (**B**) Volcano plot illustrating upregulated and downregulated genes (adjusted *p* < 0.05, |log_2_FoldChange| ≥ 1) in IMF clones compared to SF clones. Red and blue dots represent significantly upregulated and downregulated genes, respectively, while gray dots indicate non-significantly regulated genes. (**C**) Heatmap displaying the expression levels and clustering of differentially expressed genes between IMF and SF clones. Rows correspond to individual genes, and columns represent single-cell clones. Red and blue colors indicate high and low expression levels, respectively, with the color gradient reflecting the range of gene expression from high to low.

Principal component analysis (PCA) revealed that gene expression profiles from the four IMF preadipocyte clones clustered distinctly from those from the four SF preadipocyte clones (Figure 2A). Differential expression analysis identified 2002 differentially expressed genes (DEGs) between the IMF and SF preadipocyte clones (adjusted *p* < 0.05, |log2FoldChange| ≥ 1). Of these, 974 genes were upregulated and 1028 were downregulated in the IMF preadipocyte clones relative to the SF preadipocyte clones (Figure 2B,C). Among the top 10 genes with higher expression in the IMF preadipocyte clones compared to the SF preadipocyte clones were *MYOD1*, *SELP*, *MYOG*, *CHRNG*, *MYLPF*, *PDGFB*, *SELE*, *CBLN4*, *CD93*, and *MYO18B* (Table 2), while the top 10 genes expressed at lower levels in the IMF preadipocyte clones included *KCNJ5*, *SP9*, *TBX5*, *ASIP*, *LOC105605854*, *DCDC1*, *LOC101103380*, *APCDD1L*, *LOC790312*, and *CDH20* (Table 3). The full list of DEGs can be found in Table S2.

### 3.3. Expression of Preadipocyte Marker Genes in Both Intramuscular and Subcutaneous Preadipocyte Clones

**Figure 3:**
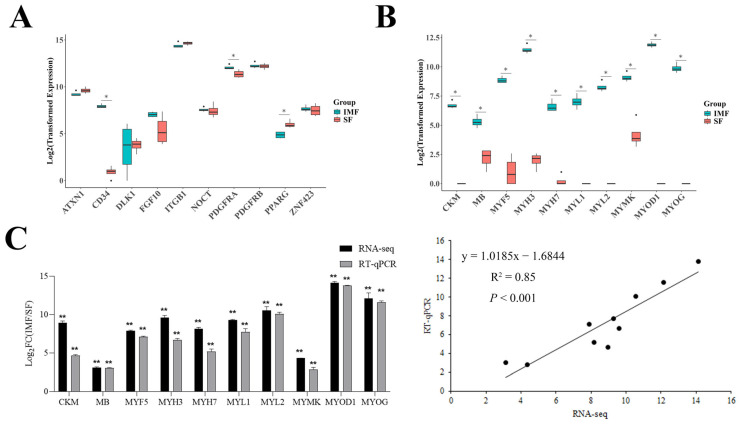
Expression levels of selected preadipocyte marker genes and muscle-specific genes in intramuscular fat (IMF) and subcutaneous fat (SF) single-preadipocyte clones. (**A**) Boxplots showing the log_2_-transformed expression levels of preadipocyte marker genes. (**B**) Boxplots showing the log_2_-transformed expression levels of muscle-specific genes. (**C**) RT-qPCR validation of expression differences in selected genes between IMF and SF preadipocyte clones determined by RNA-seq. * indicates significant difference (*p* < 0.05, *n* = 4) of gene expression between IMF and SF. ** indicates the statistical significance (*p* < 0.01, *n* = 4) of fold change in gene expression between IMF and SF clones detected by RNA-seq or RT-qPCR. Dots mean the measured genes.

Typical APC marker genes *PDGFRA* [28], as well as preadipocyte marker genes, such as *ATXN1* [29], *DLK1* [30], *PPARG* [31], *ZNF423* [32], and *FGF10* [33], were all highly expressed in both IMF and SF preadipocyte clones (Figure 3A). Additionally, *CD34* [34,35,36,37,38], *PDGFRB* [31,33,39], and *ITGB1* [33,38,40,41,42], which are known to be expressed in both adipocyte progenitors and preadipocytes, were expressed at high levels in both groups (Figure 3A). *NOCT*, previously reported as a marker of early adipogenic precursor or pro-preadipocyte cells that represent an intermediate state between APCs and preadipocytes [43], was also detected in both the IMF and SF preadipocyte clones (Figure 3A). Among these preadipocyte marker genes, *PDGFRA* and *CD34* were expressed at higher levels in the IMF preadipocyte clones than the SF preadipocyte clones, whereas *PPARG* was expressed at lower levels in the IMF preadipocyte clones (Figure 3A).

### 3.4. Expression of Many Skeletal Muscle-Specific Genes in Intramuscular but Not in Subcutaneous Preadipocyte Clones

Notably, differential expression analysis revealed that many skeletal muscle-specific genes were highly expressed in the IMF preadipocyte clones but were merely detectable in the SF preadipocyte clones. Figure 3B shows the relative expression levels of 10 skeletal muscle-specific genes in these clones. Because high-level expression of muscle-specific genes in the IMF preadipocytes was not expected, we sought to confirm this RNA-seq-based finding by quantifying the expression of 10 genes with RT-qPCR. As shown in Figure 3C, the fold changes in the expression of these genes from the IMF preadipocyte clones to the SF preadipocyte clones determined by RT-qPCR were consistent with those determined by RNA-Seq, and a strong positive correlation was observed between the fold changes detected by RNA-seq and those detected by RT-qPCR (R = 0.92, *p* < 0.001).

### 3.5. GO and KEGG Analyses Reveal Distinct Functional Signatures of Intramuscular and Subcutaneous Preadipocyte Clones

**Figure 4:**
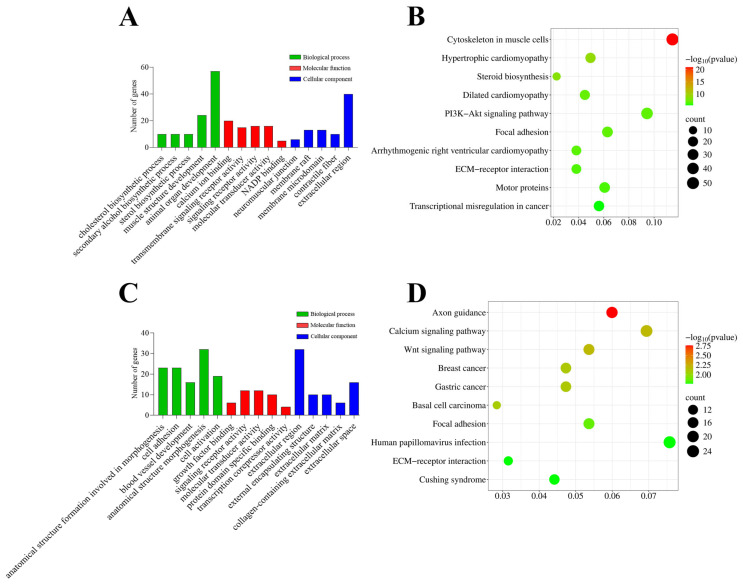
Functional enrichment analysis of genes upregulated and downregulated in intramuscular fat (IMF) single-preadipocyte clones vs. subcutaneous fat (SF) single-preadipocyte clones. (**A**) Top 5 Gene Ontology (GO) terms enriched among genes upregulated in IMF single-preadipocyte clones. (**B**) Top 10 KEGG pathways enriched among genes upregulated in IMF single-preadipocyte clones. (**C**) Top 5 Gene Ontology (GO) terms enriched among genes downregulated in IMF single-preadipocyte clones. (**D**) Top 10 KEGG pathways enriched among genes downregulated in IMF single-preadipocyte clones.

Functional enrichment analysis revealed that the genes upregulated in the IMF preadipocyte clones compared to the SF preadipocyte clones were significantly enriched in cholesterol biosynthetic process, sterol biosynthetic process, muscle structure development, calcium ion binding, neuromuscular junction, contractile fiber (Figure 4A), and pathways like cytoskeleton in muscle cells, steroid biosynthesis, focal adhesion, motor proteins, and PI3K-Akt pathway (Figure 4B). The complete results of these GO and KEGG enrichment analyses can be found in Tables S3 and S4.

In contrast, the genes downregulated in the IMF preadipocyte clones compared to the SF preadipocyte clones were enriched in biological processes such as anatomical structure formation involved in morphogenesis, cell adhesion, blood vessel development and extracellular matrix organization (Figure 4C), and pathways such as axon guidance, calcium signaling pathway, Wnt signaling pathway, focal adhesion, and ECM–receptor interaction (Figure 4D). The complete results of GO and KEGG pathway enrichment analyses of genes downregulated in the IMF preadipocytes compared to the SF preadipocyte clones can be found in Tables S5 and S6.

### 3.6. Persistent Expression of Muscle-Specific Genes in Adipocytes Differentiated from Intramuscular Preadipocytes

**Figure 5:**
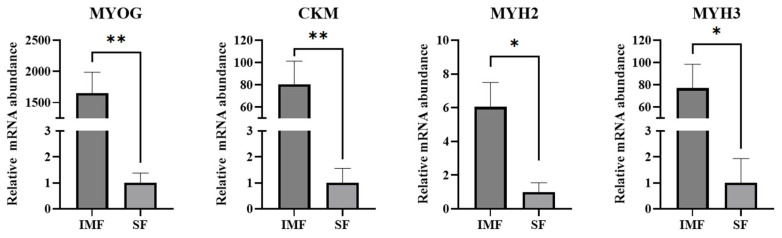
Gene expression analysis of selected muscle-specific genes (MYOG, CKM, MYH2 and MYH3) in mature adipocytes formed from intramuscular fat (IMF) and subcutaneous fat (SF) single-preadipocyte clones. * *p* < 0.05, ** *p* < 0.01 (*n* = 4).

Given the observation that the IMF preadipocytes expressed many skeletal muscle-specific genes, we further investigated whether mature adipocytes differentiated from these preadipocytes would maintain muscle-specific gene expression profile. Remarkably, mature adipocytes derived from the IMF preadipocytes maintained high-level expression of muscle-specific genes, such as *CKM*, *MYOG*, *MYH2*, and *MYH3*, which were expressed at much lower levels in mature adipocytes differentiated from the SF preadipocytes (Figure 5).

### 3.7. Bovine Satellite Cells Had Both Myogenic and Adipogenic Potentials

**Figure 6:**
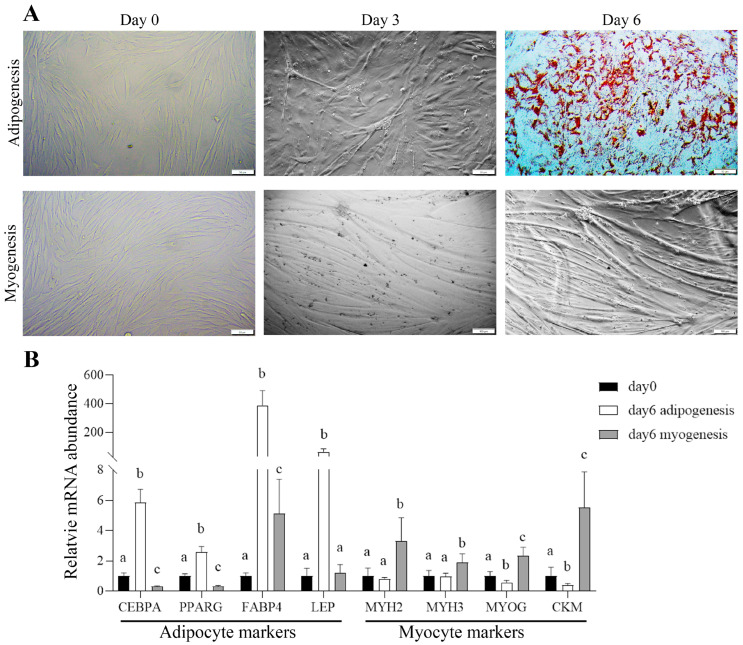
Bovine satellite cells (bSCs) have adipogenic potential in addition to myogenic potential. (**A**) Representative images of bSCs on day 0, 3, and 6 of adipogenic (**top row**) or myogenic (**bottom row**) differentiation. Scale bar: 50 μm. Cells on day 6 of adipogenic differentiation were stained with Oil Red O. (**B**) Relative expression levels of selected adipocyte marker genes (*CEBPA*, *PPARG*, *FABP4*, *LEP*) and myotube marker genes (*MYH2*, *MYH3*, *MYOG*, *CKM*) on day 0 and day 6 of adipogenic or myogenic differentiation. Bars labeled with different letters are different within genes (*p* < 0.05, *n* = 6).

Satellite cells are stem cells in postnatal skeletal muscle and can be activated by muscle injury to become myoblasts contributing to skeletal muscle regeneration and repair [44,45]. Because the IMF preadipocytes express many muscle-specific genes, we tested the possibility that the IMF preadipocytes are derived from satellite cells. To this end, we determined if bovine satellite cells (bSCs) have adipogenic potential besides myogenic potential. After 6-day culture in myogenic differentiation medium, most bSCs formed multinucleated myotubes (Figure 6A), and gene expression analysis (Figure 6B) showed significant increases in the expression of myotube marker genes, including *MYH2*, *MYH3*, *MYOG*, and *CKM*, confirming that bSCs have a robust myogenic differentiation capacity. Interestingly, after 6-day culture in adipogenic differentiation medium, many lipid droplets accumulated in bSCs (Figure 6A), accompanied by significant increases in the expression of adipocyte marker genes, including *PPARG*, *CEBPA*, *FABP4*, and *LEP* (Figure 6B). These data demonstrate that bSCs also possess adipogenic differentiation capacity.

## 4. Discussion

All adipose tissue depots harbor pools of adipocyte progenitor cells (APCs) [46,47]. Generally, researchers use the term “preadipocytes” to describe the immediate adipocyte precursor cells isolated from the SVF, which are destined solely to differentiate into mature adipocytes [13,48]. Although it is widely accepted that preadipocytes originate from APCs, significant knowledge gaps remain regarding the differences among lineage-committed preadipocytes from various adipose depots such as IMF and SF, the mechanisms guiding APCs toward a committed preadipocyte state, and even the origins of APCs. These knowledge gaps limit our ability to selectively modulate fat deposition in cattle and other economically important livestock.

In this study, we generated single-cell clones from bovine IMF and SF SVF cells, and used adipogenic differentiation assays to select those that can differentiate into mature adipocytes that are characterized by accumulating lipid droplets and expressing adipocyte marker genes such as *CEBPA*, *PPARG*, *FABP4*, and *ADIPOQ* [49]. The preadipocyte nature of selected clones was further supported by transcriptomic analysis showing that they had high expression of typical APC markers such as *PDGFRA*, preadipocyte markers such as *ATXN1*, *DLK1*, *PPARG*, *ZNF423*, and *FGF10*, and shared APC–preadipocyte markers including *CD34*, *PDGFRB*, and *ITGB1*. Notably, the early-stage marker *NOCT*, representing a transitional state between progenitors and preadipocytes [43], was also detected in both IMF and SF preadipocyte clones. *PDGFRA* is a well-known APC marker involved in preadipocyte commitment, promoting cell proliferation while inhibiting differentiation into adipocytes [33]. Among the preadipocyte-specific markers, *ATXN1* has been reported as a defining gene for preadipocytes, characterized by its elevated expression in these cells and its utility in distinguishing preadipocytes from other stromal cell types in adipose tissue [29]. *DLK1*, as an early preadipocyte surface marker, has been one of the best markers for lineage tracing, as it specifically labels early adipocyte progenitor cells and is absent in late preadipocytes and mature adipocytes [30,46]. *PPARG* is a transcription factor that plays a central role in the differentiation of preadipocytes into adipocytes, and it is essential for lipid storage and metabolism [50]. In vitro, *ZNF423* knockdown cells showed impaired adipogenesis, confirming its role as a transcriptional regulatory factor for preadipocyte commitment [32,51,52]. *FGF10* promotes preadipocyte proliferation through the Ras/MAPK pathway and plays a role in adipogenesis by influencing the expression of key regulatory factors like *CEBPB* and *PPARG* [53,54]. As for the shared markers, *CD34* is one of the most widely reported progenitor cell markers and promotes cell adhesion, migration, and differentiation [37,55]. *PDGFRB* [31] and *ITGB1* (also known as CD29) [56,57] are broadly expressed in APCs and preadipocytes and control cell proliferation and differentiation by modulating key signaling pathways and interacting with the extracellular environment. Therefore, we believe that the single-cell clones generated, selected, and characterized in this study were derived from intramuscular and subcutaneous preadipocytes.

Despite these shared features, differential expression analysis revealed clear depot-specific gene expression differences between intramuscular and subcutaneous preadipocyte clones: *PDGFRA* and *CD34* were significantly upregulated in IMF preadipocyte clones, whereas *PPARG* was more highly expressed in SF preadipocyte clones. The elevated expression of *PDGFRA* and *CD34* in IMF preadipocyte clones suggests that these cells are in an earlier progenitor state [58,59], whereas the higher expression of *PPARG* in SF clones indicates that SF preadipocytes are more advanced in the adipogenic differentiation trajectory [60]. These molecular differences partially explain the markedly slower rate of intramuscular fat accumulation compared to subcutaneous fat, as a larger proportion of progenitor cells in IMF may remain in an undifferentiated or pre-committed state rather than proceeding toward mature adipocyte formation.

However, the most striking difference in gene expression between the IMF and SF preadipocyte clones was that many muscle-specific genes were highly expressed in the IMF preadipocyte clones but not expressed in the SF preadipocyte clones. Functional enrichment analysis revealed that genes upregulated in IMF versus SF preadipocyte clones were enriched in biological processes and pathways associated with skeletal muscle, such as muscle structure development, neuromuscular junction organization, contractile fibers, cytoskeletal components of muscle cells, motor proteins, and the PI3K-Akt signaling pathway [61]. These findings indicate that IMF preadipocytes possess gene expression signatures typical of skeletal muscle cells. Myogenic gene expression pattern has been reported in brown adipocyte precursors, suggesting that brown adipocytes originate from a distinct lineage compared to white adipocytes [62]. However, in our study, IMF preadipocyte clones did not express the brown preadipocyte marker *EBF2* [63] or the brown adipocyte hallmark gene *UCP1* [64], indicating that despite expressing muscle-specific genes, they are not brown adipocyte precursors. Rather, the myogenic gene expression pattern of IMF preadipocytes suggests their developmental proximity to skeletal muscle cells. Both skeletal muscle and adipose tissue derive from mesodermal stem cells, implying a closer developmental relationship between myogenic and adipogenic lineages than with other somatic cell types [65,66]. The expression of many muscle-specific genes in IMF preadipocytes strongly suggests that they may derive from the same progenitor cells for muscle cells, possibly satellite cells. Using single-cell sequencing, Lyu et al. [67] discovered that skeletal muscle satellite cells isolated from a 2-week-old calf contain a subpopulation of cells expressing *PDGFRA*, *PPARG*, and *ZNF423*, and this subpopulation may represent IMF preadipocytes. Similarly, Yi et al. found the porcine IMF SVF contains satellite cells and myoblasts [68]. These previous findings also support a potential myogenic origin of IMF. In this study, we found that the myogenic gene expression pattern was even maintained in mature adipocytes differentiated from the IMF preadipocyte clones. The persistent expression of muscle-specific genes in mature IMF adipocytes suggests that mitochondrial activity, lipid oxidation capacity, and insulin sensitivity may be greater in IMF than in SF, as these metabolic capacities are generally higher in skeletal muscle than in adipose tissue.

In this study, we demonstrated that bSCs possess both abilities to differentiate into myotubes and adipocytes, which is consistent with a previous study [69]. These results indicate that bSCs are multipotent muscle stem cells, possessing at least both myogenic and adipogenic potential. In other words, bSCs may serve as progenitor cells for not only myoblasts, the immediate precursor cells for myocytes or myofibers, but also intramuscular preadipocytes, the immediate precursor cells for intramuscular adipocytes.

The origin of intramuscular adipocytes has been a subject of debate. An elegant study by Uezumi et al. identified *PDGFRα^+^* FAPs as the origin of adipocytes in mouse skeletal muscle, and clearly distinguished them from *Pax7^+^* satellite cells, which showed no adipogenic contribution in mice under physiological conditions [70]. This is consistent with the anatomical and metabolic characteristics of mice, which, as small mammals with high metabolic rates, deposit minimal IMF under physiological conditions [71,72,73]. While FAPs are abundant within muscle and likely contribute to the preadipocyte pool in IMF, they do not express myogenic markers such as *MYOD1* or *MYOG* [74], meaning they are clearly distinct from satellite cells. In contrast, our study suggests that in cattle, at least some IMF preadipocyte may derive from satellite cells, which is supported by previous studies showing that satellite cells can adopt adipogenic fate under regenerative or ectopic conditions [75,76,77,78]. The marked difference in adipocyte origin between these species aligns with the dramatic disparity in their IMF deposition potential. Cattle have been intensively selected for marbling, and possess a significantly greater inherent capacity for IMF deposition compared to rodents [3]. Therefore, we propose that the origin of intramuscular adipocytes is species-specific: in mice, they arise from FAPs, whereas in cattle, at least some of them are derived from satellite cells. We hypothesize that this divergence reflects not only distinct evolutionary pressures but also species-specific physiological requirements. In mice, the clear lineage separation, where FAPs are committed to adipogenesis and satellite cells to myogenesis, may facilitate rapid muscle repair. In contrast, in cattle, the ability of satellite cells to contribute to both myogenic and adipogenic lineages likely supports their enhanced capacity for marbling formation.

The finding that bovine intramuscular adipocytes may originate from satellite cells in skeletal muscle suggests that strategies promoting the adipogenic commitment of satellite cells could be used to enhance intramuscular fat deposition, or marbling, in cattle and improve beef quality.

## 5. Conclusions

Bovine intramuscular and subcutaneous preadipocytes differ significantly in gene expression profiles and signatures. Specifically, bovine intramuscular preadipocytes express many skeletal muscle-specific genes whereas bovine subcutaneous preadipocytes do not. Adipocytes differentiated from bovine intramuscular preadipocytes retain high expression of muscle-specific genes. Satellite cells from bovine skeletal muscle can differentiate into both myotubes and adipocytes. In combination, these findings suggest that bovine intramuscular and subcutaneous preadipocytes have different origins; specifically, bovine intramuscular preadipocytes may originate from muscle satellite cells.

## 6. Limitations of This Study

Several limitations of this study should be acknowledged. The preadipocyte clones analyzed in this study were derived through in vitro culture and selected based on their morphology, proliferative capacity, and differentiation potential. As a result, they may not fully represent the intramuscular or subcutaneous preadipocyte population in vivo. Moreover, while our results strongly suggest that satellite cells can give rise to intramuscular adipocytes in cattle, this notion is based solely on in vitro analyses. In vivo lineage tracing, using Cre-Lox systems or barcoding-based methods, would be required to definitively validate this finding. Understandably, applying Cre-Lox strategies in large animals like cattle presents significant technical challenges. A more practical alternative may be to trace the fate of in vitro–labeled satellite cells following transplantation.

## Figures and Tables

**Table 1:** Sequences of primers used for qPCR.

Gene ID	Primer Sequence *	GenBank Accession Number	Product Size
*HMBS*	F: CTTTGGAGAGGAATGAAGTGG R: AATGGTGAAGCCAGGAGGAA	NM_001046207.1	80
*PPARG*	F: CAGTGTCTGCAAGGACCTCA R: ATAGTGCGGAGTGGAAATGC	NM_181024.2	176
*CEBPA*	F: ATCGACATCAGCGCCTACAT R: CGGGTAGTCAAAGTCGTTGC	NM_176784.2	138
*FABP4*	F: AATTGGGCCAGGAATTTGAT R: TGGTGGTTGATTTTCCATCC	NM_174314.2	117
*ADIPOQ*	F: AGGTTGGATGGCAGGCATT R: GGACCTTCGATCCCAGTGATT	XM_024995232.2	162
*LEP*	F: GACATCTCACACACGCAGTC R: ATCGCCAATGTCTGGTCCAT	NM_173928.2	113
*MYH2*	F: CTGGCTGGAGAAGAACAAGG R: CACCGTCTGGAAAGAAGAGC	NM_001166227.1	172
*MYH3*	F: CTGGAGGAAATGAGGGATGA R: CACTCTTGAGAAGGGGCTTG	NM_001101835.2	211
*MYOG*	F: TGGGCGTGTAAGGTGTGTAA R: TATGGGAGCTGCATTCACTG	NM_001111325.1	295
*CKM*	F: TGGAGATGATCTGGACCCCA	NM_174773.4	166
	R: TTTCCCCTTGAACTCACCCG		
*MYOD1*	F: GACGGCTCTCTCTGCAACTT	NM_001040478.2	271
	R: TAGTCGTCTTGCGTTTGCAC		
*MYL1*	F: ACCCCAGCAATGAAGAGATGA	NM_001079578.1	128
	R: GAAGACACGCAGACCCTCAA		
*MYL2*	F:TCGGGGAGAAACTTAAAGGAGC	NM_001035025.2	192
	R: AGTTGCCAGTCACATCAGGG		
*MYH7*	F: CTTCAACCACCACATGTTCG	NM_174727.1	233
	R: GCTTCTGGAAGTTGCTGGAC		
*MYF5*	F: AGACGCCTGAAGAAGGTCAA	NM_174116.1	134
	R: AGCAGCTCCTGCAGACTCTC		
*MYMK*	F: GCTCGGCCATCCTCATCATT	NM_001193046.1	158
	R: GTCCCAGTCCTCGAAGAAGAA		
*MB*	F: AGTCACATGCCAACAAGCAC	NM_173881.2	108
	R: CATCAGCACCGAAGTCTGAA		

* F: forward; R: reverse.

**Table 2:** Top 10 genes upregulated in single intramuscular fat (IMF) preadipocyte-derived clones compared to single subcutaneous fat (SF) preadipocyte-derived clones.

Gene ID	Log2FC (IMF/SF)	Padj-Value	Gene Description
*MYOD1*	14.1	0.001	Myogenic differentiation 1
*SELP*	13.3	0.001	Selectin P
*MYOG*	12.1	0.001	Myogenin
*CHRNG*	12.0	0.001	Cholinergic receptor nicotinic gamma subunit
*MYL11*	11.9	0.001	Myosin light chain 11
*PDGFB*	11.8	0.001	Platelet-derived growth factor subunit B
*SELE*	11.3	0.001	Selectin E
*CBLN4*	11.2	0.001	Cerebellin 4
*CD93*	11.1	0.001	CD93 molecule
*MYO18B*	11.0	0.001	Myosin XVIIIB

**Table 3:** Top 10 genes downregulated in intramuscular fat (IMF) preadipocyte clones compared to subcutaneous fat (SF) preadipocyte clones.

Gene ID	Log2FC (IMF/SF)	Padj-Value	Gene Description
*KCNJ5*	−10.6	0.001	Potassium inwardly rectifying channel subfamily J member 5
*SP9*	−9.3	0.001	Sp9 transcription factor
*TBX5*	−8.3	0.001	T-box transcription factor 5
*ASIP*	−8.0	0.001	Agouti signaling protein
*LOC105605854*	−7.4	0.001	Uncharacterized gene
*DCDC1*	−6.7	0.001	Doublecortin domain containing 1
*LOC101103380*	−6.7	0.001	Uncharacterized gene
*APCDD1L*	−6.5	0.001	APC downregulated 1 like
*LOC790312*	−6.4	0.001	Uncharacterized gene
*CDH20*	−6.2	0.001	Cadherin 20

## Data Availability

All data generated or analyzed during this study are included in this published article and its Supplementary Information files. The sequencing data from this study has been deposited in the NCBI GEO database (https://www.ncbi.nlm.nih.gov/geo/) under accession number GSE303588.

## Supplementary Materials

The following supporting information can be downloaded at: https://www.mdpi.com/article/10.3390/cells14221807/s1, Table S1: Mapping summary of RNA-seq reads; Table S2: List of DEGs between IMF and SF preadipocyte clones. Table S3: GO terms enriched in genes upregulated in IMF preadipocyte clones. Table S4: KEGG pathways enriched in genes upregulated in IMF preadipocyte clones. Table S5: GO terms enriched in genes downregulated in IMF preadipocyte clones. Table S6: KEGG pathways enriched in genes downregulated in IMF preadipocyte clones.

## Abbreviations

The following abbreviations are used in this manuscript:

IMF	Intramuscular fat
SF	Subcutaneous fat
SVF	Stromal vascular fraction
bSCs	Bovine satellite cells
*PPARG*	Peroxisome proliferator-activated receptor gamma
*CEBPA*	CCAAT/enhancer binding protein alpha
*FABP4*	Fatty acid binding protein 4
*ADIPOQ*	Adiponectin
*MYOG*	Myogenin
*MYOD1*	Myogenic differentiation 1
*MYL2*	Myosin light chain 2
*DLK1*	Delta-like non-canonical Notch ligand 1
*PDGFRA*	Platelet-derived growth factor receptor alpha
*ZNF423*	Zinc finger protein 423
FAP	Fibro/adipogenic progenitors
APC	Adipose progenitor cells
